# Non-invasive assessment of tissue sodium content in patients with primary adrenal insufficiency

**DOI:** 10.1530/EJE-22-0396

**Published:** 2022-07-04

**Authors:** Irina Chifu, Andreas Max Weng, Stephanie Burger-Stritt, Thorsten Alexander Bley, Martin Christa, Herbert Köstler, Stefanie Hahner

**Affiliations:** 1 Division of Endocrinology and Diabetology, Department of Internal Medicine I, University Hospital of Würzburg, University of Würzburg, Würzburg, Germany; 2 Department of Diagnostic and Interventional Radiology, University Hospital Würzburg, Würzburg, Germany; 3 Comprehensive Heart Failure Center, University and University Hospital Würzburg, Würzburg, Germany; 4 Department of Internal Medicine I, University Hospital Würzburg, Würzburg, Germany

## Abstract

**Objective:**

Replacement therapy in primary adrenal insufficiency (PAI) with corticosteroids modulates sodium homeostasis. Serum sodium is, however, prone to osmotic shifts induced by several additional factors besides corticosteroids and does not always reliably reflect treatment quality. Non-osmotic tissue storage can be visualized by sodium MRI (^23^Na-MRI) and might better reflect corticosteroid activity.

**Design:**

Longitudinal study of 8 patients with newly diagnosed PAI and cross-sectional study in 22 patients with chronic PAI is reported here. Comparison was made with matched healthy controls.

**Methods:**

Using a ^23^Na-MRI protocol on a 3T scanner, relative sodium signal intensities (rSSI) to signal intensities of the reference vial with 100 mmol/L of sodium were determined in the muscle and skin of the lower calf.

**Results:**

In newly diagnosed patients, tissue rSSI (median, range) were reduced and significantly increased after treatment initiation reaching levels similar to healthy controls (muscle: from 0.15 (0.08, 0.18) to 0.18 (0.14, 0.27), *P* = 0.02; skin: from 0.12 (0.09, 0.18) to 0.18 (0.14, 0.28), *P* < 0.01). Muscle rSSI was significantly higher in patients with chronic PAI compared to controls (0.19 (0.14, 0.27) vs 0.16 (0.12, 0.20), *P* < 0.01). In chronic PAI, skin rSSI significantly correlated with plasma renin concentration.

**Conclusion:**

^23^Na-MRI provides an additional insight into sodium homeostasis, and thus the quality of replacement therapy in PAI, as tissue sodium significantly changes once therapy is initiated. The increased tissue sodium in patients with chronic PAI might be an indication of over-replacement.

## Introduction

Replacement therapy for primary adrenal insufficiency (PAI) with glucocorticoids (GC) and mineralocorticoids (MC) compensates for the existing hormone deficit and enables most patients to cope with everyday life. On closer examination, however, there is increasing evidence that a substantial number of patients suffer from physical or psychological limitations despite efforts to provide an individually adjusted medication (1, 2, 3). Currently available drugs only allow a rough imitation of physiological hormone secretion. This complicates optimal adjustment of individual replacement therapy, which should minimize the short- and long-term risks of both over- and under-replacement such as adrenal crisis or adverse cardio-metabolic outcomes (1, 4, 5).

Additionally, clinical assessment of the therapy quality relies on parameters with a low specificity such as patients’ well-being, body fluid status, serum electrolytes or plasma renin concentration (PRC) (6, 7). Not only the low specificity but also acute fluctuations of these parameters induced by trivial changes in fluid intake, body posture or time to last medication intake can mislead clinical decision-making.

Sodium homeostasis is tightly regulated by adrenal steroids, especially aldosterone. While serum sodium is susceptible to acute osmotic-driven changes due to water shifts, tissue sodium binds to proteoglycans in a water-free fashion and eludes this phenomenon (8, 9, 10).

Non-invasive assessment of tissue sodium has been made possible by sodium MRI (^23^Na-MRI). Increased muscle and skin sodium concentrations have been associated with refractory hypertension, primary aldosteronism, heart failure and even insulin resistance, suggesting that tissue sodium overload might be an independent cardiovascular risk factor (11, 12, 13, 14, 15).

Based on these observations, we pursued the question of whether changes in tissue sodium also occur in patients with primary adrenal insufficiency under standardized replacement therapy. We hypothesized that (1) patients with newly diagnosed PAI exhibit lower tissue sodium concentration compared to matched healthy controls and (2) tissue sodium concentration is restored by replacement therapy with GC and MC.

## Subjects and methods

We performed a longitudinal analysis of eight adult patients with newly diagnosed PAI recruited from the endocrinology outpatient clinic, the emergency department and the intensive care unit of the University Hospital Wuerzburg. The diagnosis of PAI was confirmed either by an insufficient cortisol response to cosyntropin (serum cortisol <20 µg/dL within 60 min after 250 µg cosyntropin i.v.) or a morning cortisol <5 µg/dL, both in combination with elevated adrenocorticotrophic hormone levels. Patients underwent ^23^Na-MRI of the calf as soon as their condition allowed lying horizontally for 30 min. The procedure was repeated within the first year after inclusion (but not earlier than 3 months after initiating replacement therapy) during a regular follow-up visit, and the results were compared to eight age-, sex- and BMI-matched healthy controls. Matching was performed according to age (±5 years) and BMI (±2 kg/m^2^) of patients at follow-up. We additionally performed a cross-sectional, case–control study in 22 adult patients with chronic PAI (>6 months) on established replacement therapy with GC and MC from our endocrinology department, who did not require dose adjustments for at least 8 weeks prior to the ^23^Na-MRI scan.

Exclusion criteria for study participants were continuous medication with drugs that can cause hyponatremia (e.g. diuretics and certain antidepressants), refractory hypertension, polydipsia (daily amount of fluid intake >3 L), diabetes insipidus and contraindications for MRI (for instance, claustrophobia, metal-containing implants, heart pacemaker and tattoos incompatible with MRI). Patients suffering from heart failure, chronic kidney disease or chronic liver disease were also excluded due to possible disease-related sodium shifts. These criteria mainly applied to the participants in the cross-sectional study. For the longitudinal analysis, newly diagnosed PAI (<1 week) was the main prerequisite for study inclusion.

A protocol deviation occurred in one case from the cross-sectional study. One patient received chronic treatment with sertraline at the time of study inclusion. Nevertheless, we decided against excluding him from the final analysis since careful review of his repeatedly documented serum sodium levels did not reveal hyponatremia at any time. Moreover, removing his data from the analysis did not have a significant impact on our results.

The following additional parameters were assessed: demographics (age, sex, BMI), medication, comorbidities, daily fluid intake (L/day), vital parameters (systolic and diastolic blood pressure and heart rate), presence of edema and biochemical parameters (serum sodium, serum potassium, serum osmolality, serum creatinine, PRC, serum copeptin levels, spot urine sodium concentration and osmolality and 24-h urinary sodium excretion).

The quality of GC and MC replacement was assessed separately by clinical scores (clinical score for assessment of replacement quality (CSARQ)) based on subjective well-being, as well as clinical and laboratory parameters as previously investigated and plotted against serum cortisol levels measured at defined time points after the morning glucocorticoid dose in 47 patients with chronic adrenal insufficiency under stable replacement therapy (6). The score significantly correlated with changes in serum cortisol across patients considered to be under-, well- and over-replaced (6). Similarly, in our study, each parameter suggesting over-replacement received 1 point, whereas for each of the parameters suggesting under-replacement, 1 point was subtracted. For GC replacement, parameters suggesting over-replacement included insomnia, recurrent infections, increased appetite, truncal obesity, weight gain >3 kg in the last 12 months, peripheral edema assessed during the clinical visit and/or reported by the patient, fasting glucose > 100 mg/dL, hypernatremia, hypokalemia and hypertensive ambulatory blood pressure (>140 mmHg systolic and/or >90 mmHg diastolic), whereas parameters suggesting under-replacement included fatigue, lack of energy and strength, myalgia, nausea, weight loss >3 kg in the last 12 months, increase of skin pigmentation, fasting glucose <60 mg/dL and/or symptoms of hypoglycemia, hyponatremia, hyperkalemia and low ambulatory blood pressure (<100 mmHg systolic and/or <60 mmHg diastolic). For MC replacement, parameters suggesting over-replacement included peripheral edema assessed during the clinical visit and/or reported by the patient, hypertensive ambulatory blood pressure (>140 mmHg systolic and/or >90 mmHg diastolic), hypernatremia, hypokalemia and PRC in the lower normal range (<10 ng/L) or suppressed (normal range: 2.7–27.8 ng/L), whereas parameters suggesting under-replacement included increased appetite for salty foods and/or salt cravings, low ambulatory blood pressure (<100 mmHg systolic and/or <60 mmHg diastolic), hyponatremia, hyperkalemia and PRC 1.5-times higher than the upper normal limit (>42 ng/L).

To assess tissue sodium content in the subjects, the calf muscle was chosen as a representative skeletal muscle. ^23^Na-MRI of the calf has proven its reliability in the past due to the good accessibility of the lower extremity and the possibility to examine the participants in supine position with dedicated hardware (11, 16, 17). Examinations were performed as proposed earlier in a cohort of patients suffering from primary hyperaldosteronism (11) in a 3 T whole-body MRI scanner (Magnetom PRISMA, Siemens). Briefly, the subjects were placed feet first in supine position. A dual-tuned ^23^Na/^1^H surface coil (Rapid Biomed, Rimpar, Germany) was used. Relevant MRI sequence parameters were repetition time 100 ms, echo time 2.01 ms, spatial resolution 3.9 × 3.9 × 20 mm^3^ and 8 averages.

To assess sodium content in the skin, parameters were adapted to obtain flat voxels with dimensions of 1.3 (AP) × 11.8 (RL) × 30 (HF) mm^3^. The flat shape of the voxels helps in reducing partial volume effects possibly impairing the results for the skin compartment.

As ^23^Na is a nucleus with a fast multi-exponential signal decay, a relevant part of the signal could not be detected with the sequence used due to the echo time of 2.01 ms. Consequently, we do not report absolute values of tissue sodium but the relative sodium signal intensity (rSSI) which is the ^23^Na signal intensity of the calf muscle relative to a reference vial containing an aqueous solution with a concentration of 100 mmol/L that was scanned with every participant. This concentration was chosen to avoid bias during calibration, as it provided a signal-to-noise ratio high enough for calibration (11, 15) while even higher concentrations might have induced image artifacts outside the vial.

The University of Wuerzburg ethics committee approved the study protocol (ethics committee approval numbers 103/18 for the longitudinal study and 217/16 for the cross-sectional study, ClinicalTrials Identifier NCT03505775). Written informed consent was obtained from all study participants before enrollment.

### Statistical analysis

Statistical analysis was performed using SPSS v. 24.0 and GraphPad Prism v. 8.4.1. Testing for normality was performed by Q-Q plots. Differences in clinical and biochemical parameters were assessed using *t* test, Mann–Whitney and Wilcoxon test. Correlations were analyzed with Pearson’s and Spearman’s correlation test. *P*-values < 0.05 were considered to be statistically significant. Results were displayed as means and s.d. or median and range, as appropriate.

## Results

### Longitudinal analysis of patients with newly diagnosed PAI

We investigated four female and four male patients. Demographic and laboratory data from study participants at first diagnosis and follow-up are displayed in Table 1. Replacement therapy with GC was initiated not earlier than 1 week prior to study inclusion (median: 3 days, range: 1–6 days).

**Table 1:** Clinical, biochemical and radiological (rSSI) characteristics of patients with PAI at first diagnosis (*n* = 8) and follow-up (*n* = 8) and matched healthy controls (*n* = 8) (longitudinal study). Data are presented as *n* (%), mean ±s.d. or as median (min, max).

	At first diagnosis	At follow-up	Healthy controls
*n*	8	8	8
Sex, male (%)	4 (50)	4 (50)	4 (50)
Age (years)	48 (23, 66)*	48 (24, 67)	45 (26, 71)
BMI (kg/m^2^)	20 (18, 26)*	23 (18, 28)**	25 (20, 30)***
Systolic blood pressure (mmHg)	115 (80, 154)	121 (110, 144)	135 (114, 149)
Diastolic blood pressure (mmHg)	60 (48, 90)	80 (60, 90)	79 (69, 90)
Serum sodium (mmol/L)	133 (99, 138)*	139 (137, 142)	141 (138, 146)***
Serum potassium (mmol/L)	5.3 ± 0.7*	4.5 ± 0.4	4.2 ± 0.4***
Creatinine (mg/dL)	1.7 ± 1.9	0.9 ± 0.2	0.9 ± 0.1
PRC (ng/L)	138 (49, 330)*	19 (8, 115)	19 (4, 38)***
Copeptin	n.a.	6.6 ± 4.2	3.3 ± 1.5
Spot urine sodium (mmol/L)	n.a.	115 ± 65	89 ± 40
rSSI muscle	0.15 (0.8, 0.18)*	0.18 (0.14, 0.27)	0.16 (0.14, 0.20)
rSSI skin	0.12 (0.9, 0.18)*	0.18 (0.14, 0.28)	0.16 (0.15, 0.25)***
Daily GC dose (mg)	30 (30, 80)	25 (15, 30)	n.a.
Daily MC dose (mg)	0 (0, 0)	0.1 (0.05, 0.1)	n.a.
CSARQ			
GC	−6 (−7, −3)*	0 (−5, 1)	n.a.
MC	−4 (−4, −1)*	0 (−1, 2)	n.a.

*P < 0.05 FD vs FU, **P < 0.05 FU vs HC, ***P < 0.05 FD vs HC.

CSARQ, clinical score for assessment of replacement quality; GC, glucocorticoid; MC, mineralocorticoid; n.a., not assessed; rSSI, relative sodium signal intensity.

Serum sodium was significantly lower whereas serum potassium and PRC were significantly higher in patients at first diagnosis compared to follow-up and to healthy controls (Table 1).

Both median muscle and skin rSSI were lower in patients at first diagnosis compared to healthy controls, but the difference reached statistical significance only for skin (skin rSSI: 0.12 (0.9, 0.18) vs 0.16 (0.15, 0.25), *P* < 0.01; muscle rSSI: 0.15 (0.8, 0.18) vs 0.16 (0.14, 0.20), *P* = 0.23) (Table 1).

Both median muscle and skin rSSI significantly increased after treatment initiation (muscle rSSI: 0.15 (0.8, 0.18) vs 0.18 (0.14, 0.27), *P* = 0.02; skin rSSI: 0.12 (0.9, 0.18) vs 0.18 (0.14, 0.28), *P* < 0.01) and reached levels similar to those measured in healthy controls (Table 1 and Supplementary Fig. 1, see section on supplementary materials given at the end of this article).

Also, the CSARQ improved significantly from −6 (−7, −3) to 0 (−5, 1), *P* = 0.01 for GC replacement and from −4 (−4, −1) to 0 (−1, 2), *P* < 0.01 for MC replacement (Supplementary Fig. 2) but did not correlate with tissue rSSI either at first diagnosis or at follow-up. The global improvement in clinical scores was driven by decreasing signs of under-replacement for each of the replacement therapies (Supplementary Figs 3 and 4).

In healthy controls, muscle rSSI correlated positively with serum osmolality (*r* = 0.8, *P* = 0.04), serum copeptin (*r* = 0.8, *P* = 0.04) and diastolic blood pressure (*r* = 0.7, *P* = 0.04). No significant correlations between tissue rSSI and clinical or laboratory parameters were found in patients.

### Cross-sectional, case–control analysis of relative sodium signal intensity in patients with CPAI

We investigated 12 male and 10 female patients with PAI. Mean time between first diagnosis and study visit was 11 ± 9 years and did not differ significantly between men and women (10 ± 11 vs 12 ± 7 years, *P* = 0.37). The hydrocortisone-equivalent dose ranged from 10 to 30 mg/day (median 20 mg/day) (Table 2). Median daily hydrocortisone dose equivalent per BMI was significantly higher in men compared to women (1.1 (0.6, 1.5) vs 0.7 (0.4, 0.9) mg, *P* = 0.03).

**Table 2:** Clinical, biochemical and radiological (rSSI) characteristics of patients with CPAI (*n* = 22) and matched healthy controls (*n* = 22) (cross-sectional study). Data are presented as *n* (%), mean ± s.d. or as median (min, max).

	CPAI	HC	*P*
*n*	22	22	
Sex, male	12 (55%)	12 (55%)	NS
Age (years)	50 (22, 71)	46 (24, 67)	NS
BMI (kg/m^2^)	26 (19, 34)	25 (21, 46)	NS
Systolic blood pressure (mmHg)	119 ± 15	127 ± 15	NS
Diastolic blood pressure (mmHg)	80 (60, 100)	79 (67, 109)	NS
Serum sodium (mmol/L)	140 ± 2	140 ± 2	NS
Serum osmolality (mosm/L)	283 ± 3	282 ± 6	NS
Serum potassium (mmol/L)	4.3 ± 0.3	4.3 ± 0.3	NS
Creatinine (mg/dL)	0.9 ± 0.2	0.8 ± 0.1	0.03
PRC (ng/L)	19 (5, 149)	13 (3, 40)	<0.01
Copeptin	5 (2, 12)	4 (2, 6)	NS
Spot urine sodium (mmol/L)	105 (34, 208)	107 (52, 274)	NS
Spot urine osmolality (mosm/L)	732 (332, 854)	740 (320, 963)	NS
24-h urine sodium (mmol/L)	187 ± 71	204 ± 104	NS
rSSI muscle	0.19 (0.14, 0.27)	0.16 (0.12, 0.20)	<0.01
rSSI skin	0.16 (0.13, 0.28)	0.16 (0.12, 0.26)	NS
Daily glucocorticoid dose (mg)	20 (10, 30)	n.a.	NS
Daily mineralocorticoid dose (mg)	0.1 (0.025, 0.15)	n.a.	NS
CSARQ			
GC	0 (−5, 2)	n.a.	NS
MC	0 (−2, 2)	n.a.	NS
Co-medication, *n*			
Vitamin D	8	1	
Vitamin B12	3	0	
l-thyroxine	20	0	
Proton pump inhibitors	5	0	
DHEA	2	0	
ASS	1	0	
Metoprolol	1	0	
Simvastatin	1	0	
Combined oral contraceptive†	1	1	
Sertraline**	1	0	

*P<0.05 CPAI vs HC **in one patient sertraline was taken for 2 years; †ethinyl estradiol/estradiol valerate + dienogest.

CPAI, patients with chronic primary adrenal insufficiency; CSARQ, clinical score for assessment of replacement quality; GC, glucocorticoid; HC, healthy controls; MC, mineralocorticoid; n.a., not assessed; NS, not significant; rSSI, relative sodium signal intensity.

Repeatedly documented sodium levels did not reveal hyponatremia at any time.

PRC (median, range) was significantly higher in patients compared to controls, both in the whole cohort (19 (5, 149) vs 13 (3, 40) ng/L, *P* < 0.01) (Table 2) and in the female subgroup (23 (7, 149) vs 10 (3, 25) ng/L, *P* < 0.01).

Muscle rSSI was significantly higher in patients compared to controls (0.19 (0.14, 0.27) vs 0.16 (0.12, 0.20), *P* < 0.01) (Table 2 and Supplementary Fig. 5). This was also separately confirmed for women (muscle rSSI: 0.19 (0.16, 0.24) vs 0.14 (0.12, 0.19), *P* < 0.01) and men (0.18 (0.14, 0.27) vs 0.17 (0.14, 0.20), *P* = 0.04). Same results were also obtained when excluding patients receiving >25 mg hydrocortisone-equivalent/day (six male patients taking 30 mg hydrocortisone/day).

Patients also exhibited significantly higher rSSI in muscle compared to skin (0.19 (0.14, 0.27) vs 0.16 (0.13, 0.28), *P* < 0.01). This difference was seen in both male and female patients but only reached statistical significance in women (0.18 (0.14, 0.27) vs 0.16 (0.14, 0.28), *P* = 0.14 for male patients and 0.19 (0.16, 0.24) vs 0.15 (0.13, 0.20), *P* < 0.01 for female patients).

For all patients, significant correlations were found between tissue rSSI and age, BMI and PRC (Supplementary Table 3). In females, rSSI correlated with the GC dose for skin (*r* = 0.64, *P* = 0.04) and with 24-h urinary sodium excretion (*r* = 0.71, *P* = 0.047) and serum potassium (*r* = −0.72, *P* = 0.019) for muscle (Supplementary Table 3).

In healthy controls, muscle and skin rSSI were positively correlated with spot urine sodium and 24-h urinary sodium excretion, respectively (Supplementary Table 4). Further positive correlations were found between skin rSSI and age (*r* = 0.55, *P* < 0.01), BMI (*r* = 0.66, *P* < 0.01) and systolic blood pressure (*r* = 0.52, *P* = 0.01).

## Discussion

We assessed for the first time tissue sodium in patients with PAI both at first diagnosis and under established replacement therapy with GC and MC. We show that tissue sodium is low in newly diagnosed patients and significantly increases under replacement therapy up to the levels of healthy controls. In the long-term, however, patients with chronic PAI display significantly higher muscle sodium levels compared to healthy controls, indicating a tissue sodium overload associated with replacement therapy.

Previous studies mainly led by Titze’s group have shown that tissue sodium reservoir expands and contracts depending on the salt intake (9). Increased muscle and skin sodium concentrations have furthermore been observed in patients with refractory hypertension, primary aldosteronism, heart failure and insulin resistance (11, 12, 14, 15, 17), most of which are associated with increased activity of GC and MC (18, 19, 20).

The lower levels of tissue sodium seen in patients with PAI at first diagnosis can be explained by increased sodium mobilization from the tissue pool in response to a constant renal sodium loss due to mineralocorticoid deficiency. Once replacement therapy is initiated, this process is reversed and tissue compartments begin restoring sodium. In the long term, however, replacement therapy might exceed physiological exposure to MC and GC, as indicated by the higher muscle sodium levels seen in our patients with chronic PAI compared to healthy controls.

Concerns about exposure to supraphysiologic glucocorticoid levels as assessed by hair cortisol and increased cardiovascular mortality and morbidity among patients with adrenal insufficiency emerged repeatedly over the last two decades (21, 22, 23, 24, 25, 26). Nevertheless, this seems to be driven by patient populations receiving higher replacement doses of GC (ranging from 25 to 50 mg/day) (23, 24, 26, 27) and MC (23), whereas lower, near-physiological doses appear to have a neutral impact on the cardiovascular risk (23).

Interestingly, the majority of our PAI patients received both GC and MC replacement doses considered to be appropriate (15–25 mg hydrocortisone-equivalent/day, 0.05–0.2 mg fludrocortisone/day) (28). Only one-third of the patients slightly exceeded the recommended upper limit of daily hydrocortisone-equivalent intake by a maximum of 5 mg (corresponding to a maximal daily dose of 30 mg). Accordingly, serum electrolytes and blood pressure did not differ from healthy controls, PRC was in the upper normal range and the clinical scores indicated a balanced replacement regimen. Nevertheless, the higher muscle sodium load seen in these patients even after excluding cases receiving >25 mg hydrocortisone/day suggests that even standard dose regimens might lead to overtreatment. Our study design, however, does not allow us to draw any robust conclusions regarding the main contributor (GC or MC) to the increased tissue sodium storage in PAI. We can only assume that MC play a significant part based on (1) the inverse correlation between skin sodium levels and PRC and (2) the higher sodium storage capacity of the muscle compared to skin, which was also reported in a rat model of MC-induced hypertension and in patients with primary aldosteronism (13, 29). However, we also found a significant positive correlation between GC dose and skin sodium levels in females. Since mineralocorticoid receptors bind both MC and GC with high affinity, it is impossible to differentiate at this point between their individual contributions.

Gender also seems to play a role in tissue sodium storage in patients with PAI. We found a positive correlation between skin sodium levels and diastolic blood pressure only in women with chronic PAI. Moreover, the significant difference in muscle sodium levels between patients with chronic PAI and healthy controls was driven by values obtained in women. Sex differences in the regulation of adrenal steroids with lower levels of cortisol and MC in premenopausal women compared to men of similar age have been reported across different settings and might further increase the risk of overtreatment in women (30, 31, 32). This fits to the observation by Skov *et al.*, demonstrating higher cardiovascular risk in females with Addisons’s disease across different tertiles of GC and MC replacement doses (23). Taking into account that the female patients in our cohort received significantly lower GC doses but similar MC doses compared to men, we could assume that the higher tissue sodium levels observed in women might be driven by MC.

Interestingly, tissue sodium correlated with 24-h sodium excretion only in healthy controls and in female patients with chronic PAI, suggesting a disruption in the physiological regulation of sodium homeostasis in adrenal insufficiency. Analyses in healthy men under a strictly controlled diet provide evidence that daily sodium excretion and changes in total-body sodium underlie aldosterone- and cortisol-dependent infradian rhythms (33). These result in periodic sodium storage independent of sodium intake (33). Failing to reproduce these physiological rhythms in PAI might explain the missing correlation between sodium storage and sodium excretion in our cohort.

Our study has several strengths and limitations. We provide for the first time an insight into tissue sodium content in patients with PAI at first diagnosis and under established replacement therapy obtained by non-invasive measurement. Including only PAI makes it, however, difficult to differentiate between the separate contributions of GC and MC. Analyzing both patients with primary and secondary AI may help to further investigate this matter. Another issue is that replacement with GC in patients with newly diagnosed PAI was initiated before the first MRI scan, as delaying treatment initiation in these patients for research purposes would have been unethical. Therefore, tissue sodium levels in this patient group do not reflect a completely treatment-naive stage. Nevertheless, we still found significantly decreased tissue sodium levels at first diagnosis. Another limitation is the lack of validation for the clinical scores assessing therapy quality. Nevertheless, these scores include clinical and biochemical parameters recommended by international guidelines and broadly used for the evaluation of the replacement therapy in adrenal insufficiency. Moreover, they were designed based on a previous study showing a significant correlation between the scoring system and serum cortisol levels following intake of the morning glucocorticoid dose in patients with chronic adrenal insufficiency. Furthermore, we did not take into consideration the phase of the menstrual cycle when examining female patients, which might induce changes in serum sodium by modulating renal sodium excretion (follicular phase) and glomerular hemodynamics (luteal phase) (34, 35, 36, 37).

One major technical limitation is the relatively long echo time of 2.01 ms in the MRI sequence used in our study. With this echo time, we surely missed a relevant part of the sodium signal because of the fast component in the multi-exponential signal decay of the ^23^Na nucleus. To take this into account, we do not provide absolute quantitative values in mmol/L but calculated a value relative to a reference vial. This approach was deeply investigated and calibrated earlier and already proved its usefulness and provided values comparable to the ones published by other groups (11, 12, 13, 17).

A great advantage of MRI is the possibility to obtain signal from voxels of rather unconventional dimensions. To obtain the sodium signal of the skin, voxels in the shape of a ‘pizza box’ were acquired to reduce the signal proportion that originates from outside the skin.

In conclusion, information on tissue sodium content provides additional insight into the effects of replacement therapy with GC and MC, as it significantly changes after initiating therapy and is not exposed to common disturbing factors such as acute osmotic shifts. Our observation of increased tissue sodium content in patients with PAI under chronic replacement therapy warrants further assessment in both patients with primary and secondary AI and correlation with clinical outcome parameters.

## Supplementary Material

Supplementary table 1. Correlations between clinical, biochemical and radiological (rSSI) characteristics of patients with CPAI (n=22)

Supplementary table 2. Correlations between clinical, biochemical and radiological (rSSI) characteristics of healthy controls (n=22)

Supplementary fig. 1. Muscle (A) and skin (B) relative sodium signal intensities (rSSI) in patients with primary adrenal insufficiency at first diagnosis (FD) and follow-up (FU) compared to matched healthy controls (HC) (n=8 per group)

Supplementary fig. 2

Supplementary fig. 3

Supplementary fig. 4

Supplementary fig. 5

## Declaration of interest

The authors declare that there is no conflict of interest that could be perceived as prejudicing the impartiality of the research reported.

## Funding

This work was supported by the Deutsche Forschungsgemeinschaft http://dx.doi.org/10.13039/501100001659 (DFG) within the CRC/Transregio 205/1 ‘The Adrenal: Central Relay in Health and Disease’ and the by the Else Kröner-Fresenius Stiftung 2019_KollegSe.03, Clinician Scientist Program ‘Rare Important Syndromes in Endocrinology’ (RISE).
