# Supplementary material for: Non-invasive assessment of tissue sodium content in patients with primary adrenal insufficiency
Source: Eur J Endocrinol. 2022 Jul 4;187(3):383–90. doi: 10.1530/EJE-22-0396 (PMC9346263; doi:10.1530/EJE-22-0396)
Supplement: Supplementary table 1. Correlations between clinical, biochemical and radiological (rSSI) characteristics of patients with CPAI (n=22) [file supplementary_table_1.pdf]

Supplementary table 1. Correlations between clinical, biochemical and radiological (rSSI) characteristics of patients with CPAI (n=22)

	**All patients (n=22)**		**Female patients (n=10)**		**Male patients (n=12)**	
	**rSSI muscle**	**rSSI skin**	**rSSI muscle**	**rSSI skin**	**rSSI muscle**	**rSSI skin**
**rSSI skin**	.32		.00		**0.76****	
**Serum sodium**	-.04	.11	-.24	.20	.13	.14
**Serum osmolality**	.35	.41	.14	.28	.52	**.59***
**Serum potassium**	.07	.12	**-.72***	-.14	.48	.32
**PRC**	-.28	**-.47***	-.32	-.21	-.27	-.54
**Copeptin**	-.05	.06	-.3	.08	.13	-.02
**Creatinine**	-.05	.31	-.57	.17	.28	.14
**Spot urine sodium**	-.18	.21	-.37	0.35	-.09	.04
**Spot urine osmolality**	**-.48***	-.41	-.14	-.40	**-.67***	-.53
**24-h urine sodium**	.09	.23	**.71***	.48	-.18	-.21
**Age**	**.60****	**.45***	**.73***	0.2	.49	**.63***
**BMI**	.02	**.44***	.02	**0.7***	-.00	.15
**Systolic blood pressure**	-.08	.19	.27	0.54	-.35	-.33
**Diastolic blood pressure**	-.25	.17	-.12	**0.7***	-.39	**-.61***
**GC dose/day**	-.25	.31	-.24	**0.64***	-.10	-.17
**GC dose/BMI**	-.2	.08	-.26	.18	-.04	-.16
**GC dose/kg**	-.10	.14	-.10	.30	.06	-.07
**GC dose/BSA**	-.22	.24	-.16	.56	-0.4	-.02.
**MC dose**	-.09	-.08	-.50	-.24	.22	.08
**MC dose/BMI**	-.11	-.24	-.52	-.54	.17	.00
**MC dose/kg**	.09	-.16	-.31	-.40	.31	.09
**MC/BSA**	-.02	-.18	-.27	-.29	.23	.09
**CSARQ GC**	.09	.13	0.33	.09	-.08	-.06
**CSARQ MC**	.28	.27	0.41	.22	.25	.26

*p<0.05, **p<0.01

rSSI=relative sodium signal intensity, PRC=plasma renin concentration, CSARQ=clinical score for assessment of replacement quality, GC=glucocorticoid, MC=mineralocorticoid, BMI=body mass index, BSA=body surface area
