# Supplementary material for: Non-invasive assessment of tissue sodium content in patients with primary adrenal insufficiency
Source: Eur J Endocrinol. 2022 Jul 4;187(3):383–90. doi: 10.1530/EJE-22-0396 (PMC9346263; doi:10.1530/EJE-22-0396)
Supplement: Supplementary table 2. Correlations between clinical, biochemical and radiological (rSSI) characteristics of healthy controls (n=22) [file supplementary_table_2.pdf]

Supplementary table 2. Correlations between clinical, biochemical and radiological (rSSI) characteristics of healthy controls (n=22)

	**Healthy controls (n=22)**	
	**rSSI muscle**	**rSSI skin**
**rSSI skin**	-.00	
**Serum sodium**	.07	-.00
**Serum osmolality**	-.22	.25
**Serum potassium**	.01	-.15
**PRC**	.18	-.4
**Copeptin**	**.55***	.27
**Creatinine**	.36	.02
**Spot urine sodium**	**.61****	.05
**Spot urine osmolality**	.09	.09
**24-h urine sodium**	.41	**.46***
**Age**	-.06	**.55****
**BMI**	.14	**.66****
**Systolic blood pressure**	-.08	**.52***
**Diastolic blood pressure**	-.13	.38

*p<0.05, **p<0.01

rSSI=relative sodium signal intensity, PRC=plasma renin concentration, BMI=body mass index
