# Supplementary material for: Non-invasive assessment of tissue sodium content in patients with primary adrenal insufficiency
Source: Eur J Endocrinol. 2022 Jul 4;187(3):383–90. doi: 10.1530/EJE-22-0396 (PMC9346263; doi:10.1530/EJE-22-0396)
Supplement: Supplementary fig. 1. Muscle (A) and skin (B) relative sodium signal intensities (rSSI) in patients with primary adrenal insufficiency at first diagnosis (FD) and follow-up (FU) compared to matched healthy controls (HC) (n=8 per group) [file supplementary_figure_1.pdf]

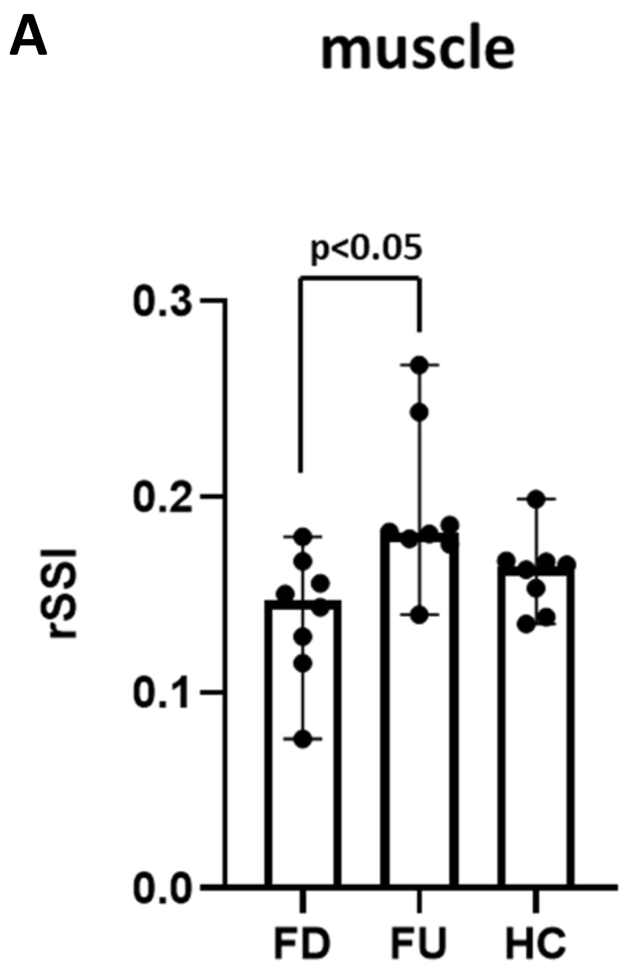

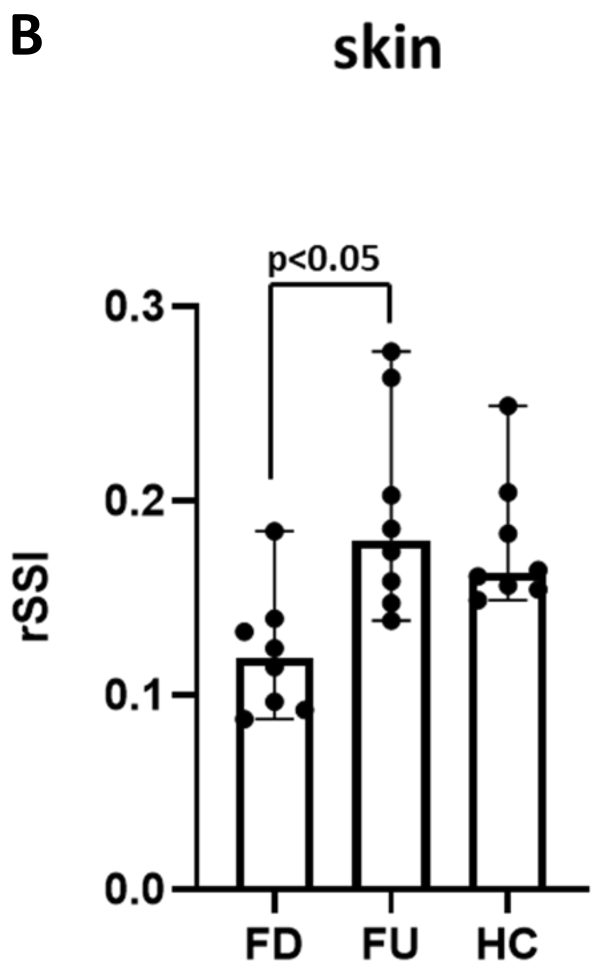

Supplementary fig. 1. Muscle (A) and skin (B) relative sodium signal intensities (rSSI) in patients with primary adrenal insufficiency at first diagnosis (FD) and follow-up (FU) compared to matched healthy controls (HC) (n=8 per group)
