# Supplementary material for: Non-invasive assessment of tissue sodium content in patients with primary adrenal insufficiency
Source: Eur J Endocrinol. 2022 Jul 4;187(3):383–90. doi: 10.1530/EJE-22-0396 (PMC9346263; doi:10.1530/EJE-22-0396)
Supplement: Supplementary fig. 2 [file supplementary_figure_2.pdf]

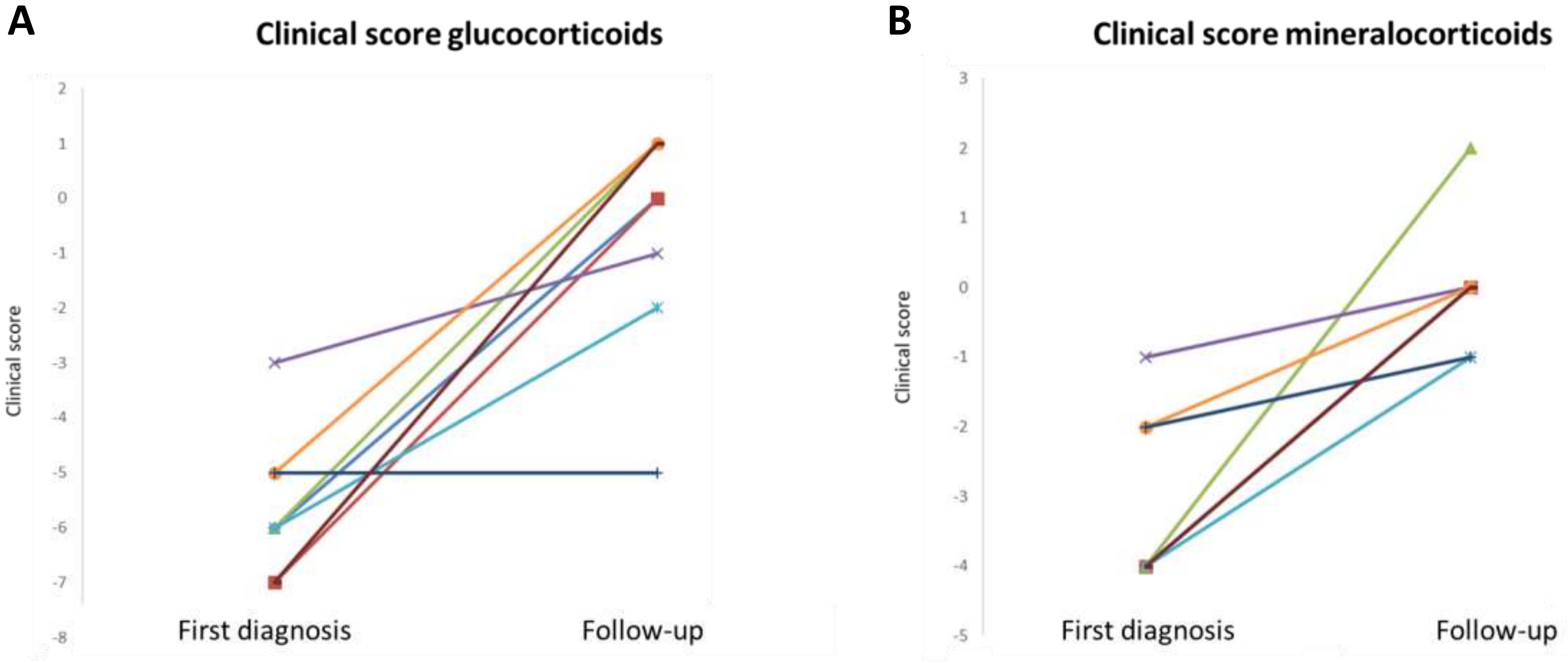

Supplementary fig. 2. Changes in clinical scores for assessment of replacement quality in patients with primary adrenal insufficiency from first diagnosis to follow-up, separately assessed for glucocorticoids (A) and mineralocorticoids (B)
