# Supplementary material for: Non-invasive assessment of tissue sodium content in patients with primary adrenal insufficiency
Source: Eur J Endocrinol. 2022 Jul 4;187(3):383–90. doi: 10.1530/EJE-22-0396 (PMC9346263; doi:10.1530/EJE-22-0396)
Supplement: Supplementary fig. 3 [file supplementary_figure_3.pdf]

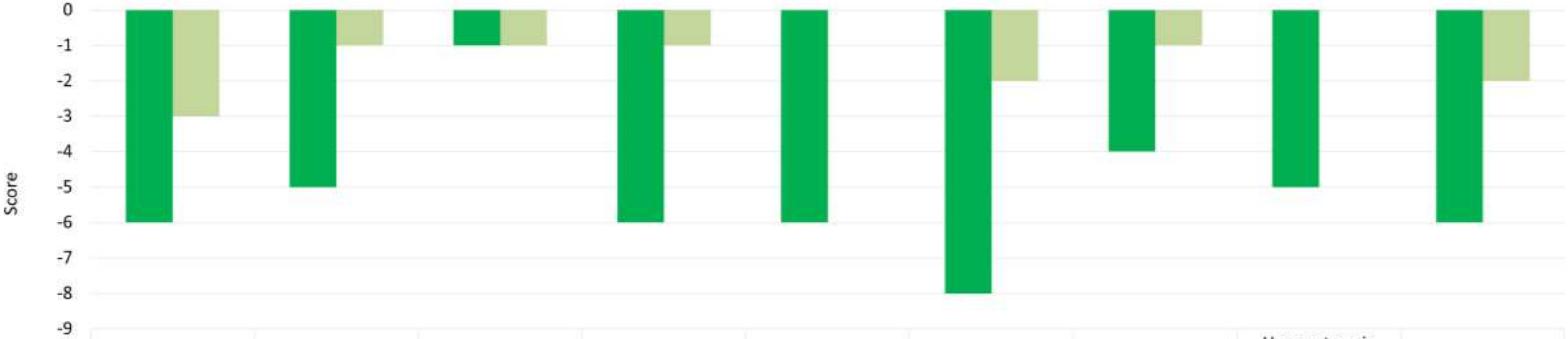

	Fatigue	Lack of energy/strength	Myalgia	Nausea	Weight loss >3kg/3 months	Hyperpigmentation	Hypoglycemia	Hyponatremia and/or hyperkalemia	Hypotension
First diagnosis	-6	-5	-1	-6	-6	-8	-4	-5	-6
Follow-up	-3	-1	-1	-1	0	-2	-1	0	-2

Supplementary fig. 3. Improvement in signs of glucocorticoid under-replacement: comparison between item scores at first diagnosis and at follow-up
