# Supplementary material for: Non-invasive assessment of tissue sodium content in patients with primary adrenal insufficiency
Source: Eur J Endocrinol. 2022 Jul 4;187(3):383–90. doi: 10.1530/EJE-22-0396 (PMC9346263; doi:10.1530/EJE-22-0396)
Supplement: Supplementary fig. 4 [file supplementary_figure_4.pdf]

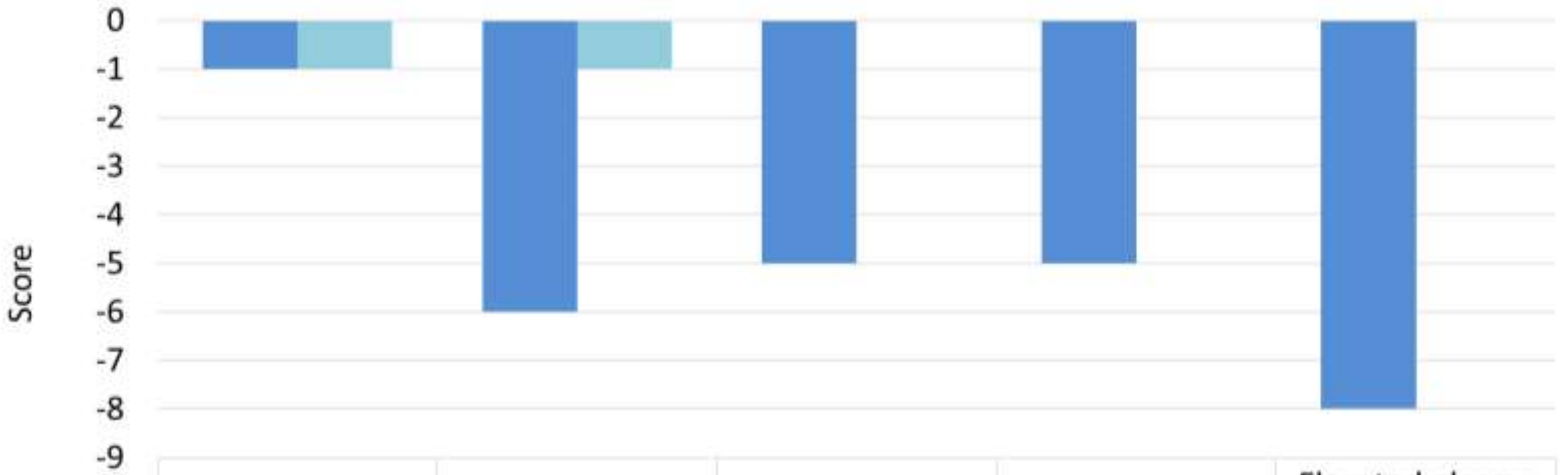

	Increased salt appetite	Hypotension	Hyponatremia	Hyperkalemia	Elevated plasma renin concentration
First diagnosis	-1	-6	-5	-5	-8
Follow-up	-1	-1	0	0	0

Supplementary fig. 4. Improvement in signs of mineralocorticoid under-replacement: comparison between item scores at first diagnosis and at follow-up
